# Combined endovascular interventions for pulmonary embolism at high altitude in Tibet

**DOI:** 10.3389/fcvm.2024.1384930

**Published:** 2024-10-11

**Authors:** Tengyan Yang, Jian Yang

**Affiliations:** ^1^ Respiratory Department, Changdu People’s Hospital of Xizang, Changdu, China; ^2^ Gastroenterology Department, The First Affiliated Hospital of Chongqing Medical University, Chongqing, China

**Keywords:** pulmonary embolism, high altitude, endovascular intervention, effectiveness, safety, Tibet

## Abstract

**Background:**

Managing pulmonary embolism (PE) at extremely high altitudes poses unique challenges due to harsh environmental conditions and limited healthcare resources.

**Method:**

This study retrospectively analyzed Tibetan PE patients in the Tibet Autonomous Region of China to evaluate the effectiveness and safety of combined endovascular interventional therapy in high-altitude areas.

**Results:**

The average altitude of long-term residence for Tibetan patients was 3,863.4 ± 317.4 m, with an average age of 62.0 ± 16.0 years, and the time from computed tomography pulmonary angiography (CTPA) diagnosis to interventional treatment averaged 2.8 ± 2.2 days. The operation time for these patients was 106.1 ± 22.2 min, and the intraoperative dose of alteplase used was 23.3 ± 5.0 mg. All 9 patients reported profound remission of dyspnea and chest pain after endovascular interventions. The heart rate (*p* < 0.05) and respiratory rate (*p* < 0.001) of all enrolled patients were significantly decreased, and the peripheral capillary oxygen saturation (SpO2) was significantly increased (*p* < 0.05) after interventions. No severe complications, such as bleeding, occurred in any patient.

**Conclusion:**

This study demonstrated the potential clinical benefits and feasibility of combined endovascular interventional therapy for treating acute PE in extreme high-altitude regions.

## 1 Introduction

Acute pulmonary embolism (PE) is a life-threatening condition characterized by a blockage of the pulmonary artery or its branches by embolic substances (thrombus, fat, or amniotic fluid) from other body parts (1). PE is the most severe clinical manifestation of venous thromboembolism (VTE) and occurs in one-third of VTE episodes, with or without deep venous thrombosis (DVT) (2, 3). VTE is the third most common acute cardiovascular syndrome globally after myocardial infarction and stroke (4). Despite advances in medical treatment, PE remains a significant healthcare burden, and its incidence is steadily increasing worldwide (5, 6).

In high-altitude regions such as the Tibetan Plateau in China, PE poses unique challenges due to the harsh environmental conditions and limited healthcare resources. Hypoxia is common among long-term residents at high altitudes, and studies have found that hypoxia leads to a pre-thrombotic state, regardless of physical activity (7). Systemic changes in adaptation to life at high altitudes create a hypercoagulable state (8). Red blood cell levels may be elevated at high altitudes, in hypoxia-related cardiopulmonary diseases, and in blood system disorders such as polycythemia vera. Red blood cells are a significant determinant of venous thrombosis size and hemorheology and, by increasing blood viscosity, become a risk factor for VT (9).

PE remains one of the most common and preventable causes of death among hospitalized patients, and the four principles of treatment include reperfusion, ensuring hemodynamic stability, achieving tissue oxygenation, and avoiding recurrence (10). Although multiple authoritative guidelines recommend thrombolysis as the first-line treatment for high-risk PE (4, 11), these drugs have been proven to be associated with increased bleeding risk. Therefore, the recommendation and application of various percutaneous endovascular interventions, including catheter-directed thrombolysis (CDT), ultrasound-assisted CDT (USCDT), and aspiration thrombectomy, and their combinations are gradually increasing (12–14).

To date, limited studies have been reported on the management of PE in high-altitude regions, particularly in the Tibetan Plateau of China. The unique physiological characteristics of high-altitude environments, such as hypoxia, low temperature, and low atmospheric pressure, may influence PE's pathophysiology, clinical presentation, and treatment effects (15, 16). Therefore, applying PE endovascular interventional therapies in high-altitude regions is clinically crucial.

Changdu is located in the eastern plateau region of China's Tibet Autonomous Region, with the highest altitude of 5,460 meters and an average altitude of 3,560 meters. In the Tibetan plateau, renowned for its five towering peaks that exceed 8,000 meters, the local population is not the sole demographic at risk of Pulmonary Embolism (PE). Travelers, mountaineers, and soldiers who venture into these high-altitude environments also face the perils associated with PE. The increased incidence of PE among these groups is attributed to various factors, including prolonged immobility, reduced oxygen saturation, and increased blood viscosity (16). Therefore, it is imperative to offer suitable and efficient medical care to mitigate the hazards posed by this life-threatening condition.

This study investigates the efficacy of combined endovascular interventional therapy in treating acute PE patients residing in Changdu. We aim to evaluate this approach's clinical outcomes, safety, and feasibility in a cohort of nine patients with acute PE who underwent endovascular interventional therapies. This study may provide valuable insights into the potential benefits of endovascular intervention for acute PE in Tibetan patients. The findings of this study will contribute to developing evidence-based guidelines for the management of PE at high altitudes and inform future clinical practice in these areas.

## 2 Materials and methods

### 2.1 Study design and setting

A retrospective analysis was carried out of Tibetan patients with acute PE who underwent a combined interventional technique between January 2023 and December 2023 in Changdu People's Hospital of Xizang. Electronic clinical records of all patients with computed tomography pulmonary angiography (CTPA) confirmed PE in Changdu People's Hospital of Xizang were retrospectively reviewed. All enrolled patients were diagnosed with PE based on Chinese standard diagnostic criteria and received combined endovascular interventional therapies, including CDT, mechanical thrombectomy, and inferior vena cava (IVC) filter implantation when necessary. Written informed consent was obtained from all the patients before each interventional procedure.

### 2.2 Inclusion criteria

• Tibetan patients with long-term residence at high altitudes over 3,400 meters;
• CTPA confirmed PE in Changdu People's Hospital of Xizang;
• Ineffective initial treatment with low-molecular heparin anticoagulation therapy at a therapeutic dose or contraindications for anticoagulant therapy;
• Resolved PE through combined endovascular interventional therapies, including CDT, mechanical thrombectomy, and IVC filter implantation in Changdu People's Hospital of Xizang.

### 2.3 Exclusion criteria

• Incomplete electronic clinical records;
• PE Diagnosis or treatment in another hospital other than Changdu People's Hospital of Xizang;
• No utilization of combined endovascular interventional therapies, including CDT, mechanical thrombectomy, and IVC filter implantation, in Changdu People's Hospital of Xizang;
• Other conditions not suitable for this study.

### 2.4 Endovascular interventional therapy for PE

**Figure 1:**
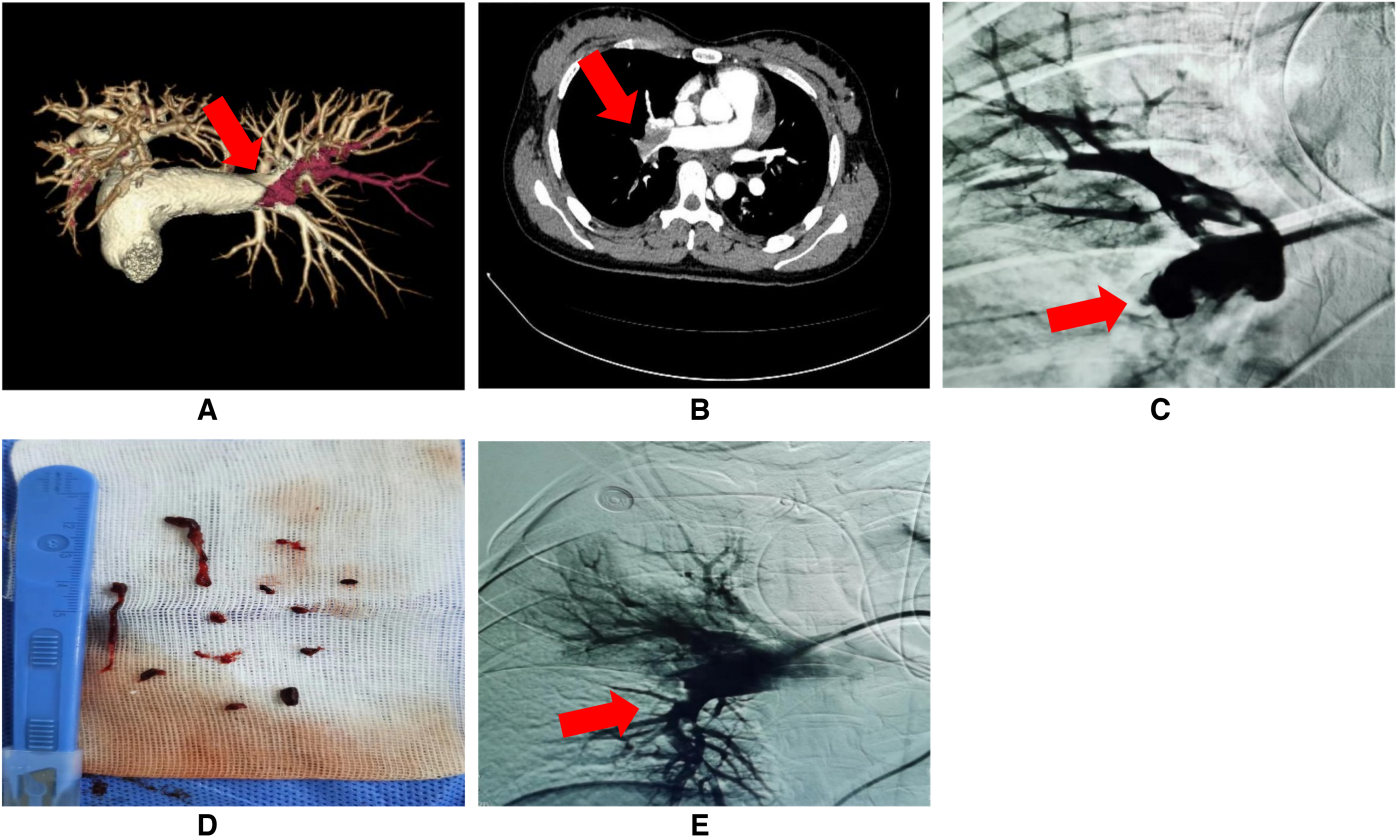
**(A**–**E)** Preoperative examination and interventional operation of PE. **(A**,**B)** Pulmonary embolism (PE) detected by preoperative CT angiography (red arrow: the site of PE); **(C)** PE confirmed by target pulmonary artery angiography (red arrow: the site of PE); **(D)** Successful thrombus aspiration; **(E)** Rechecked angiograms after interventional therapy (red arrow: the site of PE resolution).

Enrolled patients underwent preoperative CTPA to identify PE (Figures 1A,B). These patients experienced ineffective initial anticoagulation treatment with low molecular weight heparin (LWMH) at a therapeutic dose (5,000 IU i.v. q12 h), or there were contraindications for anticoagulant therapy. They voluntarily accepted combined interventional therapies, including CDT, mechanical thrombectomy, and inferior vena cava (IVC) filter implantation, at Changdu People's Hospital of Xizang. All devices and equipment used in the interventional operation were commercially available and approved by Changdu People's Hospital of Xizang. The specific interventional operation process was as follows (Supplementary Videos S1–S3).

• Routine disinfection and local anesthesia were performed around the puncture points.
• An 8F catheter-introducer kit (RS*A80K10SQ, Radifocus Introducer, Terumo Corporation, Tokyo, Japan) was inserted in the left or right femoral vein.
• Next, a 5F pigtail angiography catheter (0.035, 100 cm, Cordis Corporation, Miami Lakes, USA) was used to perform target pulmonary artery angiography to assess embolization (Figure 1C).
• A 6F long sheath (VSMP6F04, Shanghai INT Medical Instruments Corporation, Shanghai, China) was inserted under the guidance of a 0.038-inch guide wire and placed at the proximal end of the occluded blood vessel.
• An X-track™ long sheath (125 cm, MicroPort Corporation, Shanghai, China) was delivered to the thrombus for further aspiration with 10–20 mmHg negative pressure (Figure 1D). Additional contact thrombolysis by microcatheter was performed for small distal embolization. Alteplase 20–30 mg was slowly pumped for 3–5 min, and target pulmonary angiography was reevaluated(Figure 1E).
• Patients with DVT or distal venous thrombosis of IVC had thrombus filters (466-F210A, Cordis, Cashel, Ireland) implanted in their IVC. Anticoagulation with LWMH was given for 14 days (5,000 IU i.h. q12 h), and the filter was retrieved after the thrombosis disappeared by reexamination of vascular ultrasound. Rivaroxaban (15 mg bid) was then given for 21 days, followed by dose adjustment (20 mg q.d.), and continued for 3–6 months, during which regular follow-up evaluation was performed in the outpatient department of respiratory medicine.

### 2.5 Main outcomes and data sources

Heart rate, respiratory rate, blood pressure, oxygen saturation, coagulation function, and postoperative complications, including hemoptysis, dyspnea, and chest pain, were continuously monitored after intervention.

Anonymized data were collected from patients’ electronic clinical records, including age, sex, ethnicity, long-term living altitude, disease and symptoms before PE, major PE sites shown by CTPA, preoperative blood biochemical examination, vascular ultrasound, peripheral thrombolytic risk, perioperative vital signs, and postoperative complications. Records were reviewed from the time of PE diagnosis until 31 January 2024.

### 2.6 Statistical analysis

Continuous variables and categorical were expressed as means ± SD and *n* (%), respectively. A paired Student's *t*-test was used to measure continuous variables. Statistical analyses were conducted using SPSS 23.0, and statistical significance was defined as *p* < 0.05.

## 3 Results

### 3.1 Characteristics and diagnoses of enrolled patient

Nine patients with PE who were hospitalized and diagnosed at Changdu People's Hospital of Xizang between January 2023 and December 2023 were enrolled in this study. All enrolled patients underwent combined endovascular interventional therapies. The patients ranged from 33 to 73 years, with an average age of 62.0 ± 16.0 years. All patients had long-term residence in high altitudes ranging from 3,113 to 4,480 meters, with an average altitude of 3,863.4 ± 317.4 meters. The demographic characteristics and diagnoses of enrolled patients are shown in Table 1. Patients’ preoperative blood biochemical examinations and their potential risks for peripheral venous thrombolysis (PVT) are displayed in Table 2.

**Table 1:** Demographic characteristics and diagnoses of patients.

No.	Age (years)	Sex	Living altitude (meters)	First-visit department	Initial diagnosis	Final diagnosis
1	36	Male	3,880	Obstetrics	Cesarean section	PE
2	72	Male	3,413	ICU	Trauma	PE, left lower limbs DVT, trauma
3	62	Female	3,458	ER	EH	PE, both lower limbs DVT, EH
4	73	Male	3,746	Urology	UTB	PE, both lower limbs DVT, UTB
5	70	Male	3,879	IDD	PTB	PE, right lower limbs DVT, PTB
6	73	Female	4,016	Orthopaedics	Trauma	PE, right lower limbs DVT, trauma
7	68	Male	4,480	Vascular surgery Leg ulcers	PE, both lower limbs DVT, leg ulcers	
8	33	Female	4,004	Rehabilitation	ICH	PE, both lower limbs DVT, ICH
9	72	Female	3,895	Pneumology	Pneumonia	PE, both lower limbs DVT, pneumonia

ER, emergency department; IDD, infectious disease department; EH, external hemorrhoidectomy; UTB, urinary tract bleeding; PTB, pulmonary tuberculosis; ICH, intracerebral hemorrhage; PE, pulmonary embolism; DVT, deep venous thrombosis.

**Table 2:** Preoperative blood biochemical examinations and PVT risks of patients.

No.	RBC (10^12 ^/L)	Hb (g/L)	D-dimer (mg/L)	PLT (10^9 ^/L)	PVT risks
1	4.09	107↓	3.81↑	288	Incision bleeding
2	1.43↓	54↓	4.96↑	53↓	Right-sided hemothorax
3	6.59	201	16.56↑	64↓	Gastrointestinal bleeding
4	3.81	116↓	2.85↑	360	Urinary tract bleeding
5	4.64	110	1.19↑	335	Coronary artery disease
6	4,96	151	10.53↑	309	Incision bleeding
7	4.58	150	2.64↑	104	Ulcer bleeding
8	4.68	156	6.28↑	324	Intracerebral hemorrhage
9	4.14	139	2.98↑	112	Hypertension-related complications

VT, peripheral venous thrombolysis; ↑: higher than the normal value; ↓: lower than the normal value.

### 3.2 Interventional data of enrolled patients

All 9 patients received combined endovascular interventional therapy for PE, and the mean time from CTPA diagnosis of PE to endovascular interventional treatment was 2.8 ± 2.2 days (ranging from 1 to 6 days). The mean operation time and alteplase dosage were 106.1 ± 22.2 min (ranging from 70 to 140 min) and 23.3 ± 5.0 mg (ranging from 20 to 30 mg), respectively (Table 3). Patients with multiple sites of PE on intraoperative angiography (Patient No. 5, 6, 7) had an additional 10 mg of alteplase added to the standard intraoperative dose of 20 mg. Eight patients (Patients No. 1–8) underwent an IVC filter implanted for DVT or distal venous thrombosis of IVC. No significant complications such as intervention failure, major bleeding, perioperative stroke, new-onset severe respiratory failure, heart injury, cardiopulmonary arrest, or death occurred in any enrolled patients (17). Three patients (33.3%) had increased hemoptysis after the operation, which was considered to be related to the application of heparin and alteplase during the interventional procedure. No hemostatic treatment was given, and the hemoptysis gradually disappeared during postoperative observation.

**Table 3:** Interventional data of patients.

No.	Interventional timing after diagnoses (days)	Operation time(minutes)	Alteplase dosage (mg)
1	2	125	20
2	5	120	20
3	1	90	20
4	1	115	20
5	1	140	30
6	6	90	30
7	2	70	30
8	1	115	20
9	6	90	20

### 3.3 Effectiveness of endovascular interventions for PE

All 9 patients reported profound remission of dyspnea and chest pain after endovascular interventions. The heart rate (*p* < 0.05) and respiratory rate (*p* < 0.001) of all enrolled patients were significantly decreased, and the peripheral capillary oxygen saturation (SpO2) was significantly increased (*p* < 0.05) after interventions (Table 4).

**Table 4:** Comparison of patients’ vital signs during the perioperative period.

No.	HR (bpm)*		BP (mmHg)		RR (bpm)**		SpO2 (%)*^,a^	
	Pre-Op	Post-Op	Pre-Op	Post-Op	Pre-Op	Post-Op	Pre-Op	Post-Op
1	115	100	132/78	124/77	22	19	85	93
2	122	98	122/79	123/79	25	21	79	90
3	144	102	124/77	118/75	27	20	75	92
4	86	82	125/77	115/83	21	18	90	93
5	87	75	116/78	120/74	22	18	90	95
6	88	72	132/70	118/72	21	19	92	95
7	120	88	120/85	125/76	22	18	83	90
8	86	79	124/88	108/83	24	19	85	92
9	87	75	116/70	130/82	21	20	90	94

HR, heart rate; BP, blood pressure; RR, respiratory rate; SpO2, peripheral capillary oxygen saturation; Pre-Op, preoperative; Post-Op, postoperative; *p < 0.05; **p < 0.001; ^a^nasal cannula oxygen (3l /min).

CTPA reexamination 2 weeks after operation indicated the PE lesions were utterly absorbed in all enrolled patients. For 8 patients with IVC filters, DVT disappeared in 7 patients with vascular ultrasound review 2 weeks later, and their IVC filters were retrieved. One case still had DVT in his lower extremity, which may be related to the patient's mobility inconvenience after cervical spine surgery. Continued rivaroxaban (20 mg q.d.) was recommended, and the filter was removed after the disappearance of DVT on outpatient follow-up 1 month later.

## 4 Discussion

Managing PE in extreme high-altitude regions poses unique challenges due to the region's harsh environmental conditions and limited healthcare resources (15, 16, 18). Changdu People's Hospital of Xizang stands as one of the two medical institutions in the Tibet region capable of undertaking endovascular interventional therapy for PE patients. Notably, it has accomplished the highest number of endovascular interventions for PE in Tibet. This study provides valuable insights into the efficacy, safety, and feasibility of combined endovascular interventional therapy for treating PE in such environments, specifically in China's Tibet Autonomous Region. To the author's knowledge, no previous study on treating PE with endovascular interventional therapy at high altitudes in the Tibet Autonomous Region of China has been reported.

First, our findings suggest that combined endovascular interventions can be viable for acute PE patients in high-altitude areas like Changdu. Despite the physiological challenges posed by hypoxia and low atmospheric pressure, this approach demonstrated promising clinical outcomes in our cohort of nine patients. The success of these interventions is particularly noteworthy because hypoxia and low temperature can induce hypercoagulability at high altitudes by mechanisms such as upregulating transferrin, thereby increasing the risk of venous thromboembolism (19, 20). 88.9% of Tibetan PE patients in this study were accompanied by DVT, which is also consistent with the view that most PE originated from lower extremity DVT (21). Recent hospitalization, pregnancy, surgery, and infection were the pre-PE conditions in Tibetan patients in this study. These were common risk factors for PE found in previous studies (22–24). This study also found that the average age of Tibetan PE patients in high-altitude regions was 62 years old, of which 56% of patients were over 70 years old and accompanied by DVT, which was consistent with the previous study that the incidence of venous thromboembolism in people over 70 years old was significantly higher than that in other age groups (25).

Secondly, the safety profile of this study's combined endovascular interventional therapy appears acceptable, and therefore, this procedure is worthy of recommendation, considering PE's complex nature and the harsh environmental conditions. Choosing the appropriate treatment for PE patients is challenging, especially in high-altitude areas. The available methods include traditional anticoagulation, thrombolysis or surgery, and the recently developed percutaneous interventional techniques (10, 26). Many residents at high altitudes have missed their best opportunity for thrombolysis treatment due to the insidious onset of PE and relatively inconvenient transportation in plateau areas. Some other patients may have contraindications to thrombolysis similar to those enrolled in this study, so pulmonary endovascular interventional therapy is an indispensable treatment. An ideal percutaneous PE endovascular interventional technique requires high operability, effective thrombus removal, and safety with no damage to cardiopulmonary structures (27)^.^ This study used endovascular intervention, which combined CDT, mechanical thrombectomy, and IVC filter implantation to achieve PE rotation, fragmentation, aspiration, and thrombolysis, as well as accomplishing the intervention of DVT to prevent the recurrence of PE. These PE patients’ symptoms improved rapidly, and no severe complications occurred. One of the reasons for the safety of this method is the reduction of thrombolytic drugs in endovascular interventions, thus reducing the bleeding and other complications related to thrombolytic medications. The use of appropriate diameter devices (≤8F) is also an important guarantee to reduce the complications of catheter intervention techniques in our study (17). Our results further support the advantages of CDT treatment compared with standard anticoagulation therapy (14, 17). In addition, compared with the additional equipment or devices required for mechanical thrombectomy reported in the literature, the X-track™ long sheath manufactured in mainland China was used to perform intravascular thrombectomy, which was more convenient to obtain and operate without causing severe cardiopulmonary complications (13, 28). However, it is essential to acknowledge that the small sample size of this retrospective study may limit the generalizability of these findings. The currently limited sample size is partially due to the fact that only a few hospitals in the Tibet Autonomous Region of China can carry out pulmonary endovascular interventional therapies, and the duration is not long. Therefore, further research with larger cohorts is necessary to confirm the safety and efficacy of these interventions in high-altitude regions.

Thirdly, while the results of this study are encouraging and provide valuable data for the currently limited pulmonary vascular interventional therapy in the Tibetan Plateau region of China, they also highlight the need to develop evidence-based guidelines tailored to the management of PE in high-altitude areas. Hypoxia in high-altitude areas may exacerbate the symptoms of PE and increase the risk of major complications such as cardiopulmonary dysfunction and 30-day mortality (29). Similarly, low temperatures can affect the efficacy of certain drugs used to treat PE (30, 31). Therefore, such guidelines should consider the unique physiological characteristics of these regions, including hypoxia, low atmospheric pressure, and low temperature, and their potential impact on PE's pathophysiology, clinical presentation, and treatment (32). Additionally, guidelines for pulmonary embolism at high altitudes should address the practical considerations of implementing endovascular interventional therapies in areas with limited healthcare resources.

## 5 Conclusions

In conclusion, this retrospective study demonstrated the potential clinical benefits and feasibility of combined endovascular interventional therapy for treating acute PE in extreme high-altitude regions. These findings contribute to the growing knowledge of PE management in high-altitude areas and inform future clinical practice and guideline development. Further research is warranted to validate these results and to optimize the treatment strategies for PE in such unique environments.

## Data Availability

The original contributions presented in the study are included in the article/Supplementary Material, further inquiries can be directed to the corresponding author.
